# A specialized ODE integrator for the efficient computation of parameter sensitivities

**DOI:** 10.1186/1752-0509-6-46

**Published:** 2012-05-20

**Authors:** Pedro Gonnet, Sotiris Dimopoulos, Lukas Widmer, Jörg Stelling

**Affiliations:** 1 Mathematical Institute, University of Oxford, Oxford, UK; 2 Department of Biosystems Science and Engineering, , ETH Zurich, 8092 Zürich, Switzerland; 3 School of Engineering and Computing Sciences, Durham University, UK

**Keywords:** Dynamical models, ordinary differential equations, parameter sensitivities, integration

## Abstract

**Background:**

Dynamic mathematical models in the form of systems of ordinary differential equations (ODEs) play an important role in systems biology. For any sufficiently complex model, the speed and accuracy of solving the ODEs by numerical integration is critical. This applies especially to systems identification problems where the parameter sensitivities must be integrated alongside the system variables. Although several very good general purpose ODE solvers exist, few of them compute the parameter sensitivities automatically.

**Results:**

We present a novel integration algorithm that is based on second derivatives and contains other unique features such as improved error estimates. These features allow the integrator to take larger time steps than other methods. In practical applications, i.e. systems biology models of different sizes and behaviors, the method competes well with established integrators in solving the system equations, and it outperforms them significantly when local parameter sensitivities are evaluated. For ease-of-use, the solver is embedded in a framework that automatically generates the integrator input from an SBML description of the system of interest.

**Conclusions:**

For future applications, comparatively ‘cheap’ parameter sensitivities will enable advances in solving large, otherwise computationally expensive parameter estimation and optimization problems. More generally, we argue that substantially better computational performance can be achieved by exploiting characteristics specific to the problem domain; elements of our methods such as the error estimation could find broader use in other, more general numerical algorithms.

## Background

In systems biology, mathematical models often take the form of system of ordinary differential equations (ODEs). These are approximations of the underlying mechanisms such as enzyme-catalyzed biochemical reactions that are applicable when molecule numbers are sufficiently high, and when the spatial distributions of components in a cell can be neglected. More specifically, ODE models consider the rate of change in a set of states (e.g. species concentrations) as a function of the system’s current state, its inputs, and its inherent kinetic parameters that capture, for instance, affinities of molecular interactions [1].

In contrast to systems modeling in domains such as physics, however, model parameters and initial conditions for systems biology models are often not known, or they can only be roughly approximated. As few kinetic parameters can be measured directly, parametric uncertainty often prevails [2]. How the system variables depend on these system parameters can therefore be of interest, e.g. to help find parameters such that the simulated system matches some observed or desired behavior. Dependencies between system variables and parameters are captured by the local parameter sensitivities that describe to what extent the state of the system changes when parameter values are perturbed from a reference value. Formally, local parameter sensitivities comprise the set of derivatives of all system variables with respect to the system parameters. As with the state dynamics, the parameter sensitivities’ time evolution follows a system of ODEs [3].

From a computational point of view it is important to note that in all but the simplest cases, starting from a set of initial conditions, there is no direct way to compute the solutions of a system of ODEs (states or parameter sensitivities in our case) for an arbitrary time. The variables are therefore integrated numerically in small steps over time, until the desired end time is reached. Consequently, efficient and accurate numerical integration methods are critical for many applications.

The computational effort for numerical integration is linked to the system size, and over time mathematical models have become increasingly detailed to achieve better predictions. Nevertheless, even models of moderate complexity result in numerical challenges when parameter sensitivities are needed. For instance, the parameter sensitivities can be integrated naively alongside the system variables, but this implies integrating a system of size *n*_*x*_×(1 + *n*_*p*_), where *n*_*x*_ and *n*_*p*_ are the number of system variables and system parameters respectively [3].

Additionally, the solution of a system of ODEs is often used in system identification processes where global optimization or probabilistic inference are required [4,5]. In such cases, thousands, if not millions of trajectories need to be computed. Assessing the quality of the identified model, for instance with respect to the uncertainty in parameter values, again requires computing the local parameter sensitivities [6]. Although local sensitivity information can often help improve the overall estimation process, sensitivity computations are rarely included for performance reasons. Specific efficient methods exist for cases in which only scalar valued functionals are optimized [7] or oscillatory systems are considered [8]. Yet in many other cases, such as optimal control [9,10], the identification of relevant parameters [11], model reduction and simplification [12] or parameter training [13], the full parameter sensitivities need to be computed. Consequently, improvements with respect to the speed with which the original ODE systems and their parameter sensitivities can be reliably integrated may affect the entire process significantly.

These issues are not new and they concern many application domains. Considerable efforts have been invested in establishing reliable and efficient general-purpose ODE solvers for dynamic systems and—to a lesser extent—for the associated parameter sensitivities. Here, however, we are concerned with solving systems of ODEs as they typically occur in the simulation of biochemical reaction networks in systems biology [6]. We show that rather general characteristics of such systems allow for the development of application domain-oriented ODE solvers with novel numerical features (with potential broader applicability), and with superior performance compared to state-of-the-art, widely employed general-purpose solvers. To provide some context for this claim, we first briefly review key characteristics of systems biology models in the form of ODEs, and general methods for the numerical integration of ODEs.

### Dynamic models of (bio)chemical networks

When the effects of stochastic noise and of discrete molecule numbers are negligible, ODE systems can be used to describe chemical or biological reaction networks. The *n*_*x*_ time-dependent state variables *x*_*i*_(*t*,**p**), *i* = 1…*n*_*x*_, which represent the concentrations of the molecules of interest at time *t* and are usually known at some initial time *t* = *t*_0_, evolve following

(1) $$\dot{x}(t,p):=\frac{\partial x(t,p)}{\mathrm{\partial t}}=f(x(t),p)\;,\;x(t_{0},p)=x_{0}$$

where **f**(**x**(*t*),**p**) is a system of functions *f*_*i*_(**x**(*t*),**p**) modelling the conversion rate of each respective variable *x*_*i*_(*t*,**p**) at time *t*, and **p** is a vector of *n*_*p*_ system parameters.

The local parameter sensitivities with respect to some parameter *p*_*k*_ are defined as

(2) $$s_{k}(t,p):={\left(\frac{\partial x(t,p)}{\partial p_{k}}\right|}_{p=p_{0}}$$

which is the vector of the derivatives of all variables *x*_*i*_ with respect to the parameter *p*_*k*_. Similar to the dynamics in Eq. (1), the parameter sensitivities’ time evolution follows a system of ODEs given by differentiating Eq. (2) with respect to *t*:

(3) $${\dot{s}}_{k}(t,p):=\frac{\partial^{2}x(t,p)}{\partial p_{k}\mathrm{\partial t}}=\underset{:=J_{f}(x(t),p)}{\underset{︸}{\frac{\partial f(x(t,p),p)}{\partial x(t,p)}}}s_{k}(t,p)+\frac{\partial f(t,p)}{\partial p_{k}},$$

where **J**_*f*_(**x**(*t*)) is the Jacobian matrix of **f**(**x**(*t*)) with respect to **x**(*t*); note that we drop explicit dependencies on **p** to simplify notation. Initial conditions for Eq. (3) are set according to whether the initial conditions for the states in Eq. (1) depend on the parameters or not [3].

Consider, for example, the biochemical scheme of a Michaelis-Menten type enzymatic reaction

(4) $$x_{1}+x_{2}\overset{k_{1}}{\underset{k_{2}}{⇄}}\;x_{3}\;\overset{k_{3}}{\to }\;x_{1}+x_{4}$$

where *x*_1−4_ correspond to enzyme, substrate, enzyme-substrate complex, and product concentrations, respectively. With mass-action kinetics, the reaction network translates to the dynamic system

$$\dot{x}\left(t\right)=\left(\begin{array}{l} -k_{1}x_{1}x_{2}+(k_{2}+k_{3})x_{3}\\ -k_{1}x_{1}x_{2}+k_{2}x_{3}\\ +k_{1}x_{1}x_{2}-(k_{2}+k_{3})x_{3}\\ +k_{3}x_{3} \end{array}\right),\;p=\left(\begin{array}{l} k_{1}\\ k_{2}\\ k_{3} \end{array}\right).$$

Such problems are often well solved by general purpose ODE solvers, but (bio)chemical reaction networks offer a number of features that may be exploited by more specialized solvers, resulting in faster and/or more precise simulations. For instance, in enzyme kinetics, reversible association and dissociation processes are usually much faster than product formation. The resulting stiffness severely limits the types of numerical methods that can be used for ODE integration.

An opportunity for increasing solver efficiency, however, presents itself because most (bio)chemical reaction networks are only weakly interconnected. More specifically, the change in every concentration *x*_*i*_ usually depends on the concentration of very few other products. Poor connectivity is reflected in *sparse* Jacobians **J**_*f*_(**x**(*t*)), where non-zero elements correspond to interactions between components (that is, we have a correspondence to the network graph’s adjacency matrix). For the simple example Eq. (4),

$$J_{f}\left(x(t)\right)=\left(\begin{array}{llll} ∙ & ∙ & ∙ & \circ \\ ∙ & ∙ & ∙ & \circ \\ ∙ & ∙ & ∙ & \circ \\ \circ & \circ & ∙ & \circ \end{array}\right),$$

with closed and open circles indicating non-zero and zero elements, respectively. Even in this dense sub-network, the number of non-zeros $\mathrm{nn}z_{J}=5/8n_{x}^{2}$ implies that we do not need to compute a substantial number of terms to determine the Jacobian.

Many large-scale biological networks have a scale-free structure, that is, most of their nodes have few interactions, but a small number of hubs with many interactions exist [14]. This prevents an easy decomposition of a large network into subsystems that can be handled (and integrated) independently. Therefore, despite the sparsity of the Jacobian, model size remains a major issue for numerical performance.

Two more general aspects also need to be considered. Firstly, due to the growing use of abstract modeling software, the reactions and the underlying reaction equations are usually available to us as abstract models, such as smbl[15], which we can analyze and manipulate analytically. Secondly, since parametric uncertainty is abundant in biology, sensitivity analysis, i.e. the integration of the parameter sensitivities **s**_*k*_(*t*), requires particular attention. Hence, an ideal ODE solver for our application domain would efficiently and reliably handle large, stiff dynamic systems including their parameter sensitivities, and optimally exploit the systems’ non-trivial sparsity and analytic access.

### Methods for ODE integration

Almost all ODE integrators work under the assumption that the change in each variable *x*_*i*_ over time can be modeled using a polynomial in *t*. Consider the Taylor expansion of the variables **x**(*t*) around *t* = *t*_0_ to advance the system by a step of size *h*:

(5) $$x(t_{0}+h)=x(t_{0})+h\frac{\partial x}{\mathrm{\partial t}}(t_{0})+\frac{h^{2}}{2!}\frac{\partial^{2}x}{\partial t^{2}}(t_{0})+\frac{h^{3}}{3!}\frac{\partial^{3}x}{\partial t^{3}}(t_{0})+\ldots$$

If the factors *∂*^*k*^**x**(*t*) / *∂t*^*k*^ / *k*! decrease sufficiently quickly and the higher-order terms become insignificant as of some degree *n*, then we can reliably approximate the new solution by a polynomial of degree *n* in *h*. In explicit integrators, previously computed values of **x**(*t*) and the derivatives *∂***x**(*t*) / *∂t* are used to construct a polynomial **g**_*n*_(*t*) of degree *n* and to extrapolate the value of **x**(*t* + *h*) ≈ **g**_*n*_(*t* + *h*). In implicit integrators, a solution **x**(*t* + *h*) is sought such that it matches that of a polynomial **g**_*n*_(*t*) of degree *n* interpolated through previous values of **x**(*t*) and/or their derivatives, and the derivative at the solution **x**(*t* + *h*) itself. In general, implicit integrators are more accurate for stiff ODEs, where the derivatives in Eq. (5) do not decay sufficiently quickly.

Within the two larger classes, different integrators are characterized by the amount of previous values of **x**(*t*) and their derivatives which they use to approximate **x**(*t* + *h*). Table 1 lists some common integration methods; see [16] for a comprehensive review.

**Table 1:** Common ODE integration schemes and the values that are used to approximate the polynomial in Eq. (5)

**Integrator**	**Nodes, explicit^1^**	**Nodes, implicit**
Euler	**x**(*t*), $\dot{x}(t)$	**x**(*t*), $\dot{x}(t+h)$
Backward Differentiation Formula (BDF)	$\dot{x}(t)$, **x**(*t*−*h*_*k*_)	**x**(*t*), $\dot{x}(t+h)$, **x**(*t*−*h*_*k*_)
Adams-Moulton (AM)	$\dot{x}(t)$, $\dot{x}(t-h_{k})$	**x**(*t*), $\dot{x}(t+h)$, $\dot{x}(t-h_{k})$
Second-derivative rule (this work)	—	**x**(*t*), $\dot{x}(t)$, $\ddot{x}(t)$, $\dot{x}(t+h)$, $\ddot{x}(t+h)$

^1^For multi-step methods, the index *k* = 0,−1,…,−*n*.

Despite the commensurate degree of freedom in designing ODE integrators, and the number of algorithms for the numerical integration of ODEs that have been published over the past 40 years, only very few of them have found wide-spread application. Practical considerations—any method should be easily accessible to its end users, who are usually not interested in manipulating or even formulating the underlying equations themselves—are certainly major causes for this convergence [17]. However, a closer analysis of the most popular solvers for stiff ODE systems reveals another cause, namely incremental evolution.

In this area, the first major piece of software was the GEAR package [18], which by 1996 evolved into cvode[19], a part of the Sundials suite of nonlinear and differential/algebraic equation solvers [20]. The default integrators in Matlab (The MathWorks, Natick, MA) such as ode15s[21] employ similar integration rules and error estimates. Both the Sundials suite and Matlab are used increasingly in systems biology [22], but it is not evident that they are optimal for this application domain.

## Methods

### A second-derivative integrator

All ODE solvers mentioned above use only values of **x**(*t*) and $\dot{x}(t)$ to approximate **x**(*t* + *h*). Here, we differ from these methods in that we also employ the second derivatives:

(6) $$\ddot{x}(t):=\frac{\partial^{2}x(t)}{\partial t^{2}}=\frac{\partial f(x(t))}{\mathrm{\partial t}}=J_{f}(x(t))f(x(t))$$

(for notational simplicity, we will write **J**_*f*_(*t*) and **f**(*t*) instead of **J**_*f*_(**x**(*t*)) and **f**(**x**(*t*)), respectively). Note that this second derivative with respect to the time *t* should not be confused with the second-order sensitivities described and used in [9,10,23], which are the second derivatives of the system variables with respect to the system parameters.

The use of second derivatives was first suggested in [24] and later studied in detail in [25], [26] and [27], and the resulting formulas were shown to have good stability properties. A more recent study [28] reinforces the stability and potential efficiency gains for stiff systems through second-derivative methods. However, despite several published implementations [27,28], these methods have not yet found wide acceptance because, despite being *A*-stable, they are only stable at infinity if only the second derivative at *t* + *h* is used [25] (see Section S3 in Additional file 1 and Additional file 2 for details).

The second derivatives in Eq. (6) may seem somewhat clumsy and expensive to evaluate since they require the construction of the Jacobian **J**_*f*_(*t*) and the evaluation of a matrix-vector multiplication **J**_*f*_(*t*)**f**(*t*). Remember, however, that (bio)chemical reaction network models typically have sparse Jacobians. As a consequence, the cost of constructing **J**_*f*_(*t*) and of evaluating the product **J**_*f*_(*t*)**f**(*t*) grows only linearly with the number of variables and not quadratically, as the matrix-vector product would imply. Furthermore, since we usually have an abstract representation of the governing equations, we can compute each entry of $\ddot{x}(t)$ explicitly, much in the same way we evaluate the entries of **f**(*t*). For instance, the explicit second derivatives of the system Eq. (4) are:

$$\ddot{x}\left(t\right)=J_{f}\left(t\right)f\left(t\right)=\left(\begin{array}{c} -k_{1}x_{2}f_{1}-k_{1}x_{1}f_{2}+(k_{2}+k_{3})f_{3}\\ -k_{1}x_{2}f_{1}-k_{1}x_{1}f_{2}+k_{2}f_{3}\\ \;\;\;k_{1}x_{2}f_{1}+k_{1}x_{1}f_{2}-(k_{2}+k_{3})f_{3}\\ k_{3}f_{3} \end{array}\right).$$

In most cases, the evaluation of the second derivatives is not much more expensive than the evaluation of **f**(*t*).

For our second-derivative integrator, we construct an interpolating polynomial **g**_4_(*t*) of degree *n* = 4 matching **x**(*t*) and the first and second derivatives at times *t* and *t*+*h*. This implicit method requires that we find **x**(*t* + *h*) such that

$$x(t+h)=g_{4}(t+h)$$

which, expanding **g**_4_(*t* + *h*), gives us

(7) $$\;\;\;\;\;\;\;x(t+h)\;=\;x(t)+\frac{h}{2}\left[\dot{x}(t)+\dot{x}(t+h)\right]+\frac{h^{2}}{12}\left[\ddot{x}(t)-\ddot{x}(t+h)\right]\;.$$

where the right-hand side is the polynomial through **x**(*t*), $\dot{x}(t)$, $\ddot{x}(t)$, $\dot{x}(t+h)$ and $\ddot{x}(t+h)$ evaluated at *t*+*h* (this is, incidentally, the original scheme proposed in [24]). The solution to this system of equations can be computed iteratively. More specifically, we start from an initial guess $\tilde{x}(t+h)$ that is computed with an explicit formula, and use a simplified Newton’s Method:

$$\;\;\;\;\;\;x(t+h)\leftarrow x(t+h)-{\left[M(t+h)\right]}^{-1}\left(g_{4}(t+h)-x(t+h)\right)\;,$$

(8) $$M(t+h):=\frac{h}{2}{\tilde{J}}_{f}(t+h)-\frac{h^{2}}{12}{\tilde{J}}_{\mathrm{Jf}}(t+h)-I,$$

where **M**(*t* + *h*) is the Newton iteration matrix and **J**_*Jf*_(*t*) is the Jacobian of Eq. (6) with respect to **x**(*t*):

(9) $$J_{\mathrm{Jf}}(t):=\frac{\partial J_{f}(t)f(t)}{\mathrm{\partial x}}=\frac{\partial J_{f}(t)}{\mathrm{\partial x}}f(t)+{\left(J_{f}(t)\right)}^{2}$$

The Jacobians ${\tilde{J}}_{f}(t+h)$ and ${\tilde{J}}_{\mathrm{Jf}}(t+h)$ are evaluated at the initial guess $\tilde{x}(t+h)$.

Using a second-derivative scheme, the evaluation of each Newton iteration is roughly twice as expensive as for first-derivative methods of the same degree since, in addition to **f**(*t* + *h*), we must also evaluate **J**_*f*_(*t* + *h*)**f**(*t* + *h*). The advantage of this scheme, however, becomes obvious once we consider the truncation error. By replacing **x**(*t* + *h*) with the Taylor expansion around *t*, we obtain

(10) $$g_{4}(t_{n+1})-x(t_{n+1})\approx \frac{1}{720}h^{5}x^{\left(5\right)}(\xi ),\;\xi \in [t_{n},t_{n+1}]$$

for the truncation error of our second-derivative formula. For first-derivative methods of the same degree, assuming a constant step size *h*, this error is

$$\;\;\;\;\;\;\;{\mathrm{BDF}}_{4}(t_{n+1})-x(t_{n+1})\approx \frac{72}{750}h^{5}x^{\left(5\right)}(\xi ),\;\xi \in [t_{n-3},t_{n+1}],$$

$$\;\;\;\;\;\;\;{\mathrm{AM}}_{4}(t_{n+1})-x(t_{n+1})\approx \frac{19}{720}h^{5}x^{\left(5\right)}(\xi ),\;\xi \in [t_{n-3},t_{n+1}],$$

in the case of the BDF and the Adams-Moulton formula of degree four, respectively. These truncation errors are 72 times and 19 times larger than the error of our second-derivative formula (assuming the fifth derivative **x**^(5)^(*t*) is approximately constant in [*t*_*n*−3_,*t*_*n* + 1_], see Section S2 and Figure S1A in Additional file 1 and Additional file 2 for details). The large difference stems from the dependence of the interpolation error on the width of the interpolation interval, e.g. for the BDF and the Adams-Moulton formula, this interval is four times larger.

### Error estimates and step size adjustment

In any ODE integration scheme, the local error estimate and the step-size adjustment are crucial to both its accuracy and its efficiency. The step-size adjustment uses the error estimate of a previous integration step to predict the largest possible next step *h* satisfying the required tolerance. With imprecise error estimates, the step-size adjustment has to be conservative to preserve accuracy, or it risks producing an imprecise result.

In most implicit ODE solvers, the local error is either estimated from the difference between the initial estimate $\tilde{x}(t+h)$, usually computed with an explicit rule, and the final converged step **x**(*t* + *h*), or as the difference between two rules of different degree over the previous **x**(*t*) and the converged step **x**(*t* + *h*). These approaches mainly consider computational efficiency because, ideally, to estimate the error of a formula of degree *d*_1_, we need to compute a better approximation of degree *d*_2_ > *d*_1_. The difference between both converged solutions **x**_1_(*t* + *h*) and **x**_2_(*t* + *h*) can then be used to approximate the difference between the lower-degree estimate and the exact solution **x**^⋆^(*t* + *h*). However, this requires two Newton iterations to compute both solutions and if both rules have different weights for the values of $\dot{x}(t+h)$ and $\ddot{x}(t+h)$, we would need to invert or decompose two different matrices to compute a Newton iteration (Eq. (8)).

We propose a different approach that may better reconcile accuracy with computational cost. We first compute the converged lower-degree solution **x**_1_(*t* + *h*) and use it as an initial estimate for the Newton iteration of the higher-degree solution. Since we are not actually interested in the exact solution **x**_2_(*t* + *h*), but only in an approximation of the difference between the two solutions, it suffices to compute just one Newton step to get a first-oder approximation of that difference. Note that, in principle, this still requires the inversion or decomposition of a different matrix for the Newton iteration.

However, for our second-derivative solver, we can compute the second approximation as the polynomial **g**_5_(*t*) that interpolates *x*(*t*) at the same nodes as **g**_4_(*t*) plus the second to last node **x**(*t*−*h*_−1_). In this case, the weights in the Newton iteration matrix are similar. If the current step size *h* and the previous step size *h*_−1_ are equal, the weights for **J**_*f*_(*t* + *h*) and **J**_*Jf*_(*t* + *h*) are 14/31 and 2/31, respectively, which is close to the values for **g**_4_(*t*) of 1/2 and 1/12. We therefore re-use the Newton iteration matrix in Eq. (8) to compute the first approximation **x**_1_(*t* + *h*) and obtain the local error estimate

(11) $$\varepsilon :={\left[M(t+h)\right]}^{-1}\left(g_{5}(t+h)-x(t+h)\right).$$

Note that the estimate *ε* approximates the truncation error Eq. (10). Assuming that **x**^(5)^(*t*) varies only slowly between two time steps, we can compute a scaling *σ* such that the error of the next step of size *σh* is equal to a prescribed tolerance *τ*:

(12) $$\;\;\;\;\;\;\varepsilon =\frac{h^{5}}{720}x^{\left(5\right)}(\xi ),\;\tau =\frac{{\left(\mathrm{\sigma h}\right)}^{5}}{720}x^{\left(5\right)}(\xi )\;\;\Rightarrow \;\;\sigma ={\left(\frac{\tau }{\varepsilon }\right)}^{1/5}\;.$$

Note that if the assumptions on **x**^(5)^(*t*) do not hold, the error estimate in the next time step will fail, causing the step size to be reduced automatically. Furthermore, if we adjust *h* to fulfill the requested tolerance *τ* exactly, the error estimate will be larger than *τ* approximately half of the time. Therefore, in practice, we choose *σ* such that the next error will be *τ*/2. This gives us a recipe to adjust the step size from one integration step to the next and, hence, the last key ingredient of a functional second-derivative ODE solver (see Section S2 and Figure S1B of the Additional file 1 and Additional file 2 for details).

### Parameter sensitivities

For *n*_*x*_ variables and *n*_*p*_ parameters, the naive approach to sensitivity calculation implies integrating a system of *n*_*x*_×(1 + *n*_*p*_) variables and, by consequence, inverting or decomposing matrices of that size within the Newton iteration. However, the system variables **x**(*t*) do not depend on the parameter sensitivities, yet the sensitivities depend on **x**(*t*). Hence, we can, in each step, first compute the values **x**(*t* + *h*) and, once they have converged, compute the **s**_*k*_(*t* + *h*) in a separate step using the same integration rule. This staggered approach was first introduced in Caracotsis & Stewart [29], then extended by Maly & Petzold [30], and finally implemented in the Sundials cvodes ODE solver, a modified version of cvode capable of sensitivity analysis [31].

To integrate the parameter sensitivities in our second-derivative solver in a similar way, we need to compute the second derivatives

(13) $$\;\;\begin{array}{ll} {\ddot{s}}_{k}(t): & =\frac{\partial^{2}s_{k}(t)}{\partial t^{2}}=\frac{\partial }{\mathrm{\partial t}}\left(J_{f}\left(t\right)s_{k}\left(t\right)+\frac{\partial f(t)}{\partial p_{k}}\right)=J_{\mathrm{Jf}}s_{k}(t)\\ & \;+\frac{\partial \ddot{x}(t)}{\partial p_{k}}. \end{array}$$

The equation in the implicit step using the second-derivative rule in Eq. (7) for the parameter sensitivities **s**_*k*_(*t*) thus becomes

(14) $$\begin{array}{lll} \;s_{k}(t+h) & =s_{k}(t)+\frac{h}{2}\; & \;\\ & \;\times \left({\dot{s}}_{k}\left(t\right)+J_{f}\left(t+h\right)s_{k}\left(t+h\right)+\frac{\partial f(t+h)}{\partial p_{k}}\right)\; & \;\\ & \;+\frac{h^{2}}{12}\left(\;{\ddot{s}}_{k}\left(t\right)-J_{\mathrm{Jf}}\left(t\;+\;h\right)s_{k}\left(t+h\right)\;-\;\frac{\partial \ddot{x}(t\;+\;h)}{\partial p_{k}}\;\right)\;,\; \end{array}$$

which, after isolating the sole unknown term **s**_*k*_(*t* + *h*) leads to

(15) $$\begin{array}{lll} & \left[I-\frac{h}{2}J_{f}\left(t+h\right)+\frac{h^{2}}{12}J_{\mathrm{Jf}}\left(t+h\right)\right]s_{k}(t+h)=s_{k}(t)\; & \;\\ & \;+\frac{h}{2}\left({\dot{s}}_{k}\left(t\right)+\frac{\partial f(t+h)}{\partial p_{k}}\right)\; & \;\\ & \;+\frac{h^{2}}{12}\left({\ddot{s}}_{k}\left(t\right)-\frac{\partial \ddot{x}(t+h)}{\partial p_{k}}\right).\; \end{array}$$

We then have two alternatives to compute **s**_*k*_(*t* + *h*): either iteratively using Newton’s method to solve Eq. (14), or directly by inverting or decomposing the matrix on the left-hand side of Eq. (15). The iterative approach via Eq. (14) is equivalent to the one suggested in [30], and we can re-use the inverted or decomposed iteration matrix used to compute the variables **x**(*t* + *h*) in Eq. (8). However, one has to re-evaluate the Jacobians at each iteration to compute ${\dot{s}}_{k}(t)$ and ${\ddot{s}}_{k}(t)$ because ${\tilde{J}}_{f}(t+h)$ and ${\tilde{J}}_{\mathrm{Jf}}(t+h)$ are evaluated at the initial estimate $\tilde{x}(t+h)$, and not at the converged solution **x**(*t* + *h*). To compute **s**_*k*_(*t* + *h*) directly, which corresponds to the original approach in [29], we need to re-compute the Jacobians and the inverse or decomposition of the left-hand side of Eq. (15). For small *n*_*x*_, however, this extra matrix computation may be advantageous over running the Newton iteration for each parameter *p*_*k*_.

Furthermore, if the Jacobians do not vary significantly over time, they can be re-used as the matrices ${\tilde{J}}_{f}(t)$ and ${\tilde{J}}_{\mathrm{Jf}}(t)$ for the Newton iteration of the next step. Such an approach offers an advantage if the cost of running an additional *n*_*p*_ Newton iterations to compute the parameter sensitivities iteratively outweighs the cost incurred by the slower convergence due to using older Jacobians in the next step. In our second-derivative integrator, we therefore compute the parameter sensitivities directly as per Eq. (15).

### Framework for conversion of SBML models

In order to generate the matrices **J**_*f*_(*t*) and **J**_*Jf*_(*t*), as well as the second derivative $\ddot{x}(t)$ automatically, we established a framework that automatically translates arbitrary models from the standard SBML format [15] to Matlab functions or C-language code. The framework also generates routines to compute the parameter derivatives *∂***f**(*t*)/*∂***p** and $\partial \ddot{x}(t)/\partial p$ necessary for the parameter sensitivity computations. This conversion, which needs to be done only once per model, exploits the sparsity of the corresponding matrices by generating compact expressions for their non-zero entries only, making them efficient to evaluate. It uses the Matlab Symbolic Toolbox to manipulate, differentiate and simplify the resulting expressions automatically (see Sections S1.3 and S1.4 in the Additional file 1 and Additional file 2 for details).

## Results and discussion

### Implementation and testing

We implemented the second-derivative ODE integrator as odeSD in Matlab and as odeSD_mex_ in the C programming language, using the Matlab mex interface with calls to the LAPACK and BLAS libraries for the linear algebra operations. Both solvers provide an interface similar to that of the Matlab default integrators. Additionally, a native C-language version, odeSD_c_, was implemented for use outside of the Matlab programming environment. All implementations could operate on any type of ODE-based model, but the overall implementation is targeted to systems biology models in standard SBML format, for which we developed an automatic model conversion framework (see Methods). The implementation details are described in Sections S1.1 and S1.2 of the Additional file 1 and Additional file 2.

We compared our algorithm against three integrators which use Newton’s method to compute each implicit step:

1.Matlab’s default integrator for stiff systems, ode15s[21], which uses a 5-point Numerical Differentiation Formula (NDF), a more stable variant of the BDF integration rules; it is used as the default integrator in SBToolbox2 [22],

2.the cvode integrator from the Sundials suite [20] which employs variable-order BDFs of up to degree 4; it is the integrator used in the SBML ODE Library (SOSlib) [32], and

3.the radau5 integrator, a fifth-order three-stage implicit Runge-Kutta method for stiff systems described in [33] and implemented in Matlab [34].

The Matlab interface supplied by the sundialsTB toolbox [35] served to run the Sundials integrators which are implemented in C.

For performance evaluation, we selected a number of curated systems biology models from the BioModels database [36] (Table 2). This set comprises systems of different sizes (up to the largest models available in the database) and characteristics, namely convergence to steady-state and (stiff) oscillatory behavior. Note that all models have sparse Jacobians, as is evident from the number of non-zero elements *nnz*(*J*_*f*_).

**Table 2:** Systems biology test models and their key characteristics, namely number of states (*n*_*x*_), number of parameters (*n*_*p*_), number of non-zeros of the Jacobians *nnz*(**J**_*f*_), integration time interval (*t*), and biological system described by the model

**Model**	***n*_*x*_**	***n*_*p*_**	***nnz*(J_*f*_)**	***nnz*(J_*Jf*_)**	***t***	**Comments**
Hornberg *et al.*[37]	8	19	22	32	[0,100]	Steady state, ERK^1^ phosphorylation and kinase/phosphatase control.
Kholodenko *et al.*[38]	23	51	137	372	[0,100]	Steady state, short term signaling by the EGF receptor.
Singh *et al.*[39]	66	109	323	846	[0,35 000]	Steady state, IL-6 signal transduction in hepatocytes.
Borisov *et al.*[40]	90	136	468	2156	[0,1 000]	Steady state, insulin-EGF network interactions in mitogenic signaling.
Ung *et al.*[41]	200	314	956	4038	[0,4 000]	Steady state, regulation of EGFR endocytosis and EGFR-ERK signaling by crosstalk.
Elowitz & Leibler [42]	6	8	12	18	[0,1 000]	Oscillatory, synthetic network of transcriptional regulators.
Leloup & Goldbeter [43]	10	48	30	52	[0,300]	Oscillatory, circadian oscillations of PER and TIM proteins in *Drosophila*.
Wolf *et al.*[44]	13	40	47	99	[0,300]	Oscillatory, autonomous metabolic oscillations in continuous culture of *S. cerevisiae*.
Goldbeter & Pourquié [45]	20	73	47	76	[0,300]	Oscillatory, segmentation clock by crosstalk of Notch, Wnt, and FGF pathways.
Xie & Kulasiri [46]	24	49	57	107	[0,100]	Oscillatory, circadian rhythms in *Drosophila* with interlocked feedback loops.

^1^Abbreviations used: ERK = extracellular regulated kinase; EGF = epidermal growth factor; IL-6 = interleukin 6; FGF = fibroblast growth factor.

### Integrator performance without parameter sensitivities

**Figure 1:**
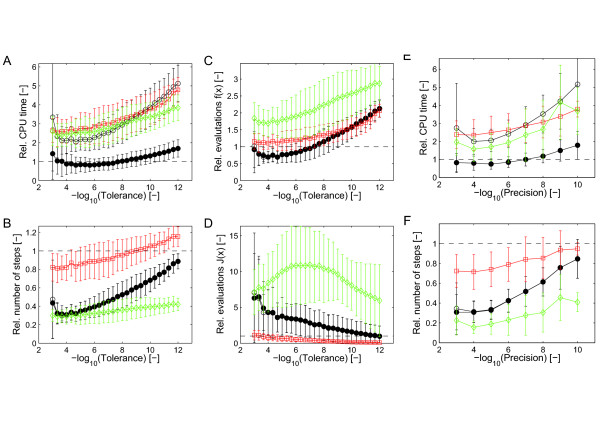
**Performance comparison without parameter sensitivities.** Performance comparison for integration of ODE-based systems biology models without parameter sensitivities. (**A**) Computation times, (**B**) number of integration steps, (**C**) number of r.h.s. evaluations **f**(**x**), and (**D**) number of evaluations of the Jacobian **J**_*f*_(**x**) as a function of the relative numerical tolerance. Symbols specify the integrators ode15s (open red squares), radau5 (open green diamonds), odeSD (open black circles), and odeSD_mex_ (filled black circles), respectively. Performance metrics are normalized to the corresponding measures for cvode and averaged (mean ± std.) over all models, which were integrated over the time spans given in Table 2; the dashed line indicates performance equal to cvode. (**E**) Computation times and (**F**) number of integration steps as a function of numerical precision (see main text for definition) in analogy to (**A**) and (**B**).

The results of the performance comparison without sensitivity analysis for a wide range of integration tolerances are summarized in Figure 1 (see Additional file 1: Figures S2-3 for details). The average computational times for our integrator were comparable (odeSD_mex_ vs. cvode) to, or slightly lower (odeSD vs. ode15s or radau5) than those of the first-derivative solvers (Figure 1A), except for low numerical tolerances. Importantly, the second-derivative integrator required approximately half as many steps as ode15s or cvode (Figure 1B), despite these three integrators using rules of the same degree of precision. The radau5 integrator used less steps than odeSD, but it computes two additional intermediate steps per full step. The smaller number of steps in odeSD is due to a combination of both the smaller truncation error of the second-derivative rule and the better accuracy of the improved error estimate. For more detailed results and discussion, see Section S3 and Figure S2 of the the Additional file 1 and Additional file 2.

**Figure 2:**
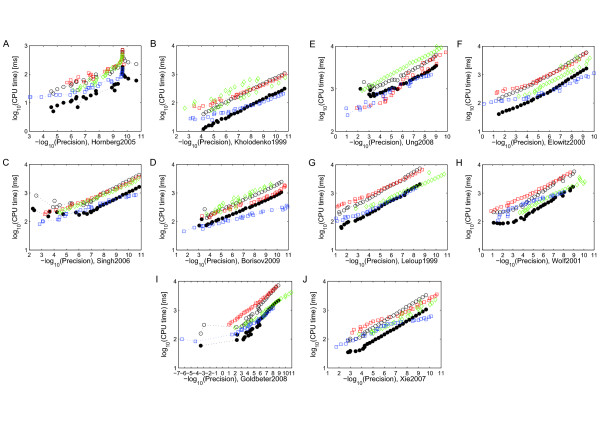
**Precision/work diagrams without parameter sensitivities.** Precision-work diagrams for integration without parameter sensitivities. (**A-J**) Computation times for the individual models (see X-axis for model specifications) as a function of precision using odeSD (open black circles), odeSD_mex_ (filled black circles), ode15s (red squares), radau5 (green diamonds), and cvodes (blue squares). All models were integrated for the time spans shown in Table 2.

To assess the relative accuracy of odeSD, we compared the results of all models computed with different relative tolerances with an ‘accurate’ reference solution computed using radau5 with the relative tolerance set to 10^−15^, analogously to the precision/work tests in [26]. The measured precision for each model and integrator is the maximum relative error in the final step for each state larger than machine precision in the reference solution. Figure 2 shows these results for all models in Table 2. These results are summarized in Figure 1E with the CPU time averaged over all models and the precision binned to the closest power of 10. Overall, without computing the parameter sensitivities, the new integrator is competitive, in terms of accuracy and efficiency, with highly optimized state of the art solvers for hard numerical problems in our application domain.

### Integrator performance with parameter sensitivities

The performance comparison with sensitivity calculations requires two additional considerations: Since ode15s and radau5 do not provide any special functionality for computing parameter sensitivities, we used an augmented system of size *n*_*x*_×(*n*_*p*_ + 1) including an analytic sparse Jacobian for each model, and the sensitivities **s**_*k*_(*t*), *k*=1…*m* were integrated alongside the system variables. cvodes, the sensitivity analysis-enabled version of cvode from the sundials package, uses a simultaneous integrator based on the method of Maly & Petzold [47]. Optionally, the staggered integrator of Feehery *et al.*[30] can be selected, but this did not produce better results.

In all cases, parameter sensitivities were integrated to the same precision as the system variables. As with the integration without sensitivities, precision/work diagrams were computed for all models with sensitivities, omitting the cases in which ode15s failed completely.

**Figure 3:**
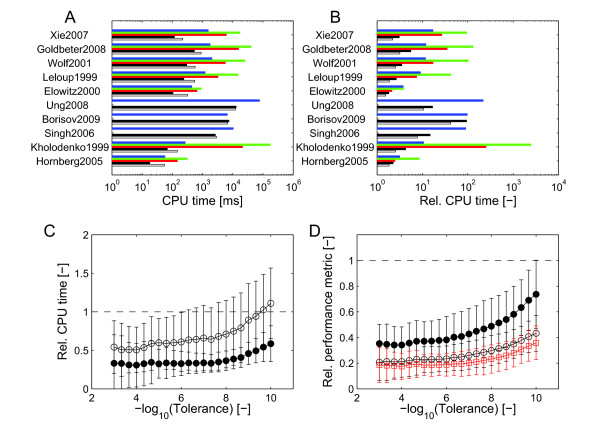
**Performance comparison with parameter sensitivities.** Performance comparison with parameter sensitivities. (**A**) Computation times for the individual models listed in Table 2 with relative tolerance of 10^−6^ using odeSD (white bars), odeSD_mex_ (black), ode15s (red), radau5 (green), and cvodes (blue). Due to the explosion in compute time, the three largest steady-state models were not evaluated with ode15s and radau5. (**B**) CPU times with sensitivity calculation as in (**A**) relative to CPU times without sensitivity calculation. (**C**) Average, normalized (see below) CPU times with sensitivities as a function of the relative numerical tolerance for odeSD (open circles) and odeSD_mex_ (filled circles) relative to cvodes. (**D**) Relative numbers of integration steps (open black circles), of function evaluations **f**(**x**) (filled black circles), and of evaluations of the Jacobians **J**_·_(·) (open red squares) for odeSD_mex_ compared to cvodes, respectively. In all cases, model and sensitivity equations were integrated for the time spans shown in Table 2. Performance metrics in (**C**, **D**) are normalized to the corresponding measures for cvodes and averaged (mean ± std.); the dashed line indicates performance equal to cvodes.

As one detailed example, Figure 3A shows the computation times for a relative tolerance of 10^−6^. In most cases, the compute times with sensitivities are substantial (see also Additional file 1: Figure S3 for other tolerances). For ode15s and radau5, the size of the augmented system quickly becomes a problem as the solution of the linear system of equations in the Newton iteration scales cubically with the number of variables. In terms of the additional effort for the sensitivity computation, we note that the second-derivative integrators are more efficient, often increasing the compute time only two- or three-fold, whereas the overhead is substantial for cvodes (Figure 3B). The results for cvodes are best explained if we keep in mind that whenever the right-hand side **f**(·) is evaluated, the algorithm also computes ${\dot{s}}_{k}(t)$, *k*=1…*n*_*p*_, which, as per Eq. (3), requires an evaluation of the Jacobian **J**_*f*_(·). As a consequence, our second-derivative integrator outperforms the Sundials solver when implemented in C using the Matlab mex interface (odeSD_mex_), and even in native Matlab (odeSD) for all larger models. Note that the higher compute times for odeSD_mex_ vs. odeSD in some cases are the result of a more refined handling of near-singular matrices by Matlab in the latter.

**Figure 4:**
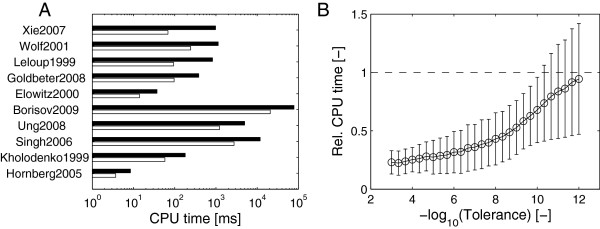
**Performance comparison of C-language integrators with parameter sensitivities.** Performance comparison with parameter sensitivities of the C-language version odeSD_c_ with cvodes using the automatically generated, compiled C-language right-hand side and Jacobian functions. (**A**) Computation times for the individual models listed in Table 2 with relative tolerance of 10^−6^ using odeSD (white bars) and cvodes (black). (**B**) Average, normalized (see below) CPU times as a function of the relative numerical tolerance for odeSD relative to cvodes. In all cases, model and sensitivity equations were integrated for the time spans shown in Table 2. Performance metrics in (**B**) are normalized to the corresponding measures for cvodes and averaged (mean ± std.); the dashed line indicates performance equal to cvodes.

In order to obtain results independent of any potential inefficiencies of the Matlab interface, the same performance analysis was run using odeSD_c_ and cvodes with natively compiled C-language functions for the right-hand sides. The results of this comparison are summarized in Figure 4 (see Additional file 1: Figure S4 in the Additional file 1 and Additional file 2 for the detailed precisionwork diagrams).

**Figure 5:**
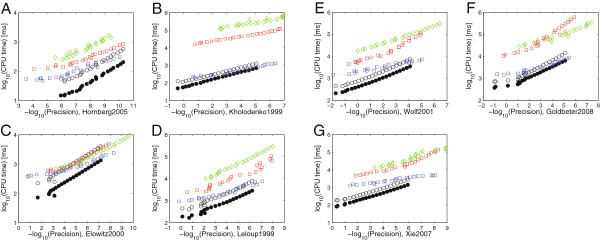
**Precision/work diagrams with parameter sensitivities.** Precision-work diagrams for integration with parameter sensitivities. (**A-G**) Computation times for all models for which the systems dynamics were solved with all ODE integrators (see X-axis for model specifications) as a function of precision using odeSD (open black circles), odeSD_mex_ (filled black circles), ode15s (red squares), radau5 (green diamonds), and cvodes (blue squares). The models were integrated for the time spans shown in Table 2.

The higher efficiency of odeSD, odeSD_mex_ and odeSD_c_ holds also for averages over all models and for a wide range of numerical tolerances (Figure 3C). Except for high-precision integration, we achieve approximately two to three-fold speed-ups. These general findings also hold when compute time is assessed as a function of numerical precision (Figure 5).

To explain the performance, consider that although odeSD and odeSD_mex_ also evaluate the Jacobians in each step, both the much smaller number of steps required (Figure 3D), which is of no particular advantage when the parameter sensitivities are not computed, and the re-use of the Jacobians for sensitivity computations lead to significantly shorter execution times since substantially fewer evaluations of **f**(·) and of the Jacobians **J**_(·)_(·) are needed (Figure 3D). Since the latter dominates the integration cost, all three versions of odeSD outperform cvodes in all but the smallest systems. These results are not a consequence of the sparsity of the systems per se, but of the more precise integration rule which can be computed efficiently thanks to sparsity. The better error estimate and the computation of local parametric sensitivities are therefore particular strengths of our ODE solver based on second derivatives.

## Conclusions

We have presented an integrator for ODE systems resulting from the modeling of chemical and biological reaction networks, which are often stiff and sparse. For the realistic systems biology models tested, the new integrator outperforms commonly used state of the art integrators when parameter sensitivities are required. It is competitive in integrating the system equations alone, despite limitations for specific models near the steady state. The improvements with respect to sensitivity calculations are critical for many applications to drive highly compute-intensive (global) optimization and estimation processes.

The improvements themselves are due to a combination of several factors: The more accurate second-derivative rule allows us, in combination with a better error estimate, to take larger steps, which in turn allows us to reduce the number of otherwise expensive sensitivity calculations. The re-use of the Jacobians from the sensitivity calculations further reduces the total computational costs. Although each integration step is more expensive than in first-derivative methods, due to the additional second-derivative information that needs to be computed, far less steps are required in total, resulting in a more efficient method.

To be of practical relevance for applications in systems biology, odeSD and odeSD_mex_ are accessible via Matlab interfaces^a^, and we plan to make them more easily available through integrated modeling environments such as the SBToolbox2 [22] and COPASI [48], e.g. via the native C-language interface. To accelerate larger optimization and estimation processes, further efficiency improvements through the use of sparse matrix routines and by more elaborate step size control schemes are possible.

In terms of numerical algorithms, to our knowledge, this is the first practical application of a second-derivative integration method with good performance. Key, novel features of our integrator, such as a more precise error estimator and direct computation of parameter sensitivities with re-use of the Jacobians, may well be suited for other problems or types of integrators. Importantly, while our integrator has been developed for (bio)chemical and reaction networks, it is still quite general in targeting stiff and sparse ODE systems. Overall, we feel that a lot is to be gained by adapting general algorithms to specific problem domains, and that results from the work on specific problems can spill over to the broader field.

### Availability

The integrator (Matlab and C-language versions) and the model conversion framework are available via http://www.csb.ethz.ch/tools.

## Endnotes

^a^We note that it is not possible to execute odeSD_mex_ in the 64-bit Windows version of Matlab R2010a as well as in 64-bit Linux versions prior to R2010a in our testing environments, for reasons of memory allocation problems in Matlab.

## Competing interests

The authors declare that they have no competing interests.

## Authors’ contributions

PG developed the second-derivative integration scheme for the parameter sensitivities and the new error estimate, as well as the final Matlab, mex and C-language versions. SD developed the automatic generation of the right-hand sides. LW implemented and tested the first versions of the integrator. JS designed and conducted the experiments. Both PG and JS wrote the manuscript, which was read and approved by all authors.

## Supplementary Material

Additional file 1 **Supplementary Text and Figures for A Specialized ODE Integrator for the Efficient Computation of Parameter Sensitivities**. Click here for file

Additional file 2 **Supplementary Source Code for A Specialized ODE Integrator for the Efficient Computation of Parameter Sensitivities**. Click here for file
