# Supplementary material for: A specialized ODE integrator for the efficient computation of parameter sensitivities
Source: BMC Syst Biol. 2012 May 20;6:46. doi: 10.1186/1752-0509-6-46 (PMC3522561; doi:10.1186/1752-0509-6-46)
Supplement: Additional file 1 — Supplementary Text and Figures for A Specialized ODE Integrator for the Efficient Computation of Parameter Sensitivities. [file 1752-0509-6-46-S1.pdf]

# Supplementary Material for A Specialized ODE Integrator for the Efficient Computation of Parameter Sensitivities

Pedro Gonnet[1,2], Sotiris Dimopoulos[2] , Lukas Widmer[2] and Jörg Stelling*[2]

[1]Mathematical Institute, University of Oxford, Oxford, UK
[2]Department of Biosystems Science and Engineering, ETH Zurich, 8092 Zürich, Switzerland

Email: Pedro Gonnet - gonnet@maths.ox.ac.uk; Sotiris Dimopoulos - sotiris.dimopoulos@bsse.ethz.ch; Lukas Widmer - luwidmer@ethz.ch; Jörg Stelling*- joerg.stelling@bsse.ethz.ch;

*Corresponding author

## S1 Implementation Details

For all computations with `odeSD` and $\texttt{odeSD}_{\texttt{mex}}$, we used Matlab version 7.10.0.499 (R2010a), 64-bit, `sundialsTB` v.2.4.0, a Matlab implementation of `radau5` [1], and `gcc` 4.1.2 under Red Hat 4.1.2-48 Linux on an IBM x3850 M2 server with 64 GB RAM and Intel Xeon X7350 CPUs at 2.93 GHz. For the performance evaluations, all processes were run in single-threaded mode.

The native c-language $\texttt{odeSD}_{\texttt{c}}$ was compiled using `gcc` 4.4.5 and linked against the Atlas library 3gf for the LAPACK and CBLAS routines. The comparison of $\texttt{odeSD}_{\texttt{c}}$ and `cvodes` was run on a 64-bit 2.2 GHz AMD Opteron running Red Hat 4.4.5-6 Linux.

To achieve reliable timing data, all models were integrated 10 times. Evaluation numbers for derivatives and Jacobians were obtained by designing appropriate function wrappers. Numerical precision was evaluated as the maximal relative deviation between a reference solution (obtained with `radau5` at relative numerical tolerance of $10^{-15}$) and the approximation with variable tolerance at the end time of integration over all system states. States with values below machine precision were excluded from this analysis. To determine the average performance as a function of numerical precision, precisions were binned (with centers as shown in the corresponding figures), and performance indicators were averaged over these intervals and over all models. For the automatic function generation from SBML models, we used Symbolic Toolbox V5.2 and the current version of SBtoolbox2 that supports all features of SBML Level 1 and 2.

### S1.1 Details of the Matlab Integrator

To retain a certain level of compatibility, `odeSD` uses the same calling sequence as the default integrators in Matlab:

```
[ T, X, TE, YE, IE, S, SE ] = odeSD ( f, t, x0, opts, params, varargin{:} )
```

where the return value `T` is a vector containing the time steps and `X` is a matrix whose columns are the variables at each time step. The $\texttt{odeSD}_{\texttt{mex}}$ integrator uses the same interface, yet is called using `odeSD_wrapper` instead of `odeSD`. The return value `S` contains a three-dimensional array containing the values of the parameter sensitivities at each time step. The return values `TE`, `YE`, `IE` and `SE` contain, analogously to the Matlab integrators, the data at points where user-specified events (see the `Events` option below) were triggered. The parameter sensitivities are only computed if the return value `S` is requested. The parameter `t` is a vector of length at least two containing the time interval over which to integrate. If `t` is of length larger than two, the values of $\mathbf{x}(t)$ are only stored and returned for those

times. Otherwise, all computed values of $\mathbf{x}(t)$ are returned. The optional parameter `opts` contains an ODE-options structure as is available for the other Matlab integrators in which the options `RelTol`, `AbsTol`, `NormControl`, `NonNegative`, `OutputFcn`, `OutputSel`, `Refine`, `Stats`, `InitialStep`, `MaxStep`, `Events` and `Jacobian` can be set. The optional parameter `params`, which is required when the output `S` is requested, contains a vector of parameter values to be passed to the right-hand side `f` for sensitivity analysis and the variable parameters `varargin{:}` are passed to the right-hand side `f`.

The right-hand side `f` is expected to be either a function or a handle on a function of the form

```
[ dxdt, d2xdt2, J_f, J_Jf, dfdp, d2fdpdt ] = f ( t, x, params, varargin{:} )
```

where `dxdt` and `d2xdt2` are the first and second derivatives $\dot{\mathbf{x}}(t)$ and $\ddot{\mathbf{x}}(t)$ respectively. The output arguments `J_f` and `J_Jf` are the Jacobian matrices of the first and second derivatives, respectively. The outputs `dfdp` and `d2fdpdt`, which are only required if the parameter sensitivities need to be computed, are matrices containing the derivatives of the first two outputs with respect to the parameters where the $k$th column of `dfdp` contains the derivatives of the variables with respect to the $k$th parameter. Similarly, the function specified with the `Jacobian` option in `opts` should have the form

```
[ J_f, J_Jf ] = J ( t, x, params, dxdt, varargin{:} ).
```

where the first derivative `dxdt` is supplied as it may simplify the computation of the Jacobians.

If no initial step size is specified in `opts`, a default value of 1/100th of the integration interval is assumed, which is repeatedly scaled by a factor of 0.7 until the extrapolated initial guess $\tilde{\mathbf{x}}(t_0 + h) = \mathbf{x}_0 + h\mathbf{f}(\mathbf{x}_0) + h^2/2\mathbf{J}(\mathbf{x}_0)\mathbf{f}(\mathbf{x}_0)$ satisfies all non-negativity constraints specified by the option `NonNegative`.

In every step, the algorithm computes an initial guess $\tilde{\mathbf{x}}(t + h)$ by constructing a BDF over the last three points and extrapolating to the new time $t + h$. In case no previous steps are available, a linear extrapolation using the zeroth and first derivative at $t$ is used, and if only one step is available, the zeroth and first derivatives of the last two steps are employed.

If the step size has changed from the previous step, the iteration matrix in (8) is reassembled using the most recent copies of the Jacobians $\tilde{\mathbf{J}}_f(t + h, \tilde{\mathbf{x}}(t + h))$ and $\tilde{\mathbf{J}}_{Jf}(t + h, \tilde{\mathbf{x}}(t + h))$ and is decomposed using an LU factorization. If after two iterations of Newton's method the solution diverges or is not expected to converge within five steps (see [2] for details on how convergence is estimated), the Jacobians are re-evaluated, the iteration matrix is reassembled and decomposed, and the iteration is restarted. If the Jacobians are up to date, the iteration is abandoned and the step size $h$ is reduced by a factor of 0.7.

Once the Newton iteration has converged, the error is approximated as in (11). The maximum relative component-wise error is used to compute the scaling factor $\sigma$ for the next step as in (12) with a tolerance $\tau$ of half of the requested tolerance. Note that since the scaling $\sigma$ is computed to achieve half of the required tolerance, a step only fails if $\sigma < (1/2)^{1/5} \approx 0.87$. If the error estimate is below the requested tolerance, the algorithm proceeds to compute, if requested, the parameter sensitivities, as per (15). For the parameter sensitivities, the same error estimator and step size scaling are applied as for the system variables.

The fifth-degree rule used in the error estimate of both the system variables and the parameter sensitivities is

$$
\begin{aligned}
g_5(t+h) = {} & \frac{1}{6h_{-1}^2 + 10h^2 + 15hh_{-1}} \\
& \left( \frac{6h_{-1}^5 + 10h_{-1}^3 h^2 + 15hh_{-1}^4 + h^5}{h_{-1}^3}\mathbf{x}(t) - \frac{h^5}{h_{-1}^3}\mathbf{x}(t - h_{-1}) \right. \\
& + h\frac{8hh_{-1}^3 + 3h_{-1}^4 + 6h^2h_{-1}^2 - h^4}{h_{-1}^2}\dot{\mathbf{x}}(t) + h(3h_{-1}^2 + 7hh_{-1} + 4h^2)\dot{\mathbf{x}}(t) \\
& \left. + h^2\frac{3h^2h_{-1} + h^3 + 3hh_{-1} + h_{-1}^3}{2h_{-1}}\ddot{\mathbf{x}}(t) - h^2(h^2 + h_{-1}^2 + 2hh_{-1})\ddot{\mathbf{x}}(t+h) \right)
\end{aligned}
$$

where $h_{-1}$ is the size of the previous step.

### S1.2 Details of the C-language integrator

The interface of the native c-language `odeSD`$_c$ is similar to that of the Matlab version. The calling sequence, as defined in `odeSD.h` is

```
int odeSD ( f_t *f , dfdx_t *dfdx , int nr_tspan , double *tspan , int N ,
    const double *x0 , struct odeSD_opts *opts , int nr_params ,
    const double *params , void *varargin , double **t_out , double **x_out ,
    double **s_out );
```

where `f_t` is a function of the type

```
int f ( double t , const double *x , const double *p , void *varargin , double *f ,
    double *dfdt , double *J , double *dJ , double *dfdp , double *d2fdtdp );
```

and `dfdx_t` is a function of the type

```
int dfdx ( double t , const double *x , const double *f , const double *p ,
    void *varargin , double *J , double *dJ , double *dfdp , double *d2fdtdp );
```

The functions `f` and `dfdx` compute the first derivatives and Jacobians, respectively. The resulting vectors and matrices are stored in column-major order in the output variables `f` (first derivative), `dfdt` (second derivative), `J` (Jacobian of first derivative), `dJ` (Jacobian of second derivative), `dfdp` (derivative of `f` with respect to the parameters) and `dfdp` (derivative of `dfdt` with respect to the parameters). These variables point to memory allocated by `odeSD` and are `NULL` if the value is not required. The values `x` and `p` contain the `N` system variables and `nr_params` parameters respectively. The variable `varargin` passed to `odeSD` is passed on to `f` and `dfdx`.

The output variables `t_out`, `x_out` and `s_out` are analogous to the return values of the Matlab integrator and will contain pointers to these arrays in column-major ordering, allocated by `odeSD` using `malloc`.

The argument `opts` is a pointer to a structure of the type `odeSD_opts` defined in `odeSD.h` which contains options analogous to those of `odeset` in Matlab. The global variable `odeSD_opts_default` contains the default settings. The arguments `x0` and `params` contain the initial system variables and the parameters respectively and `tspan` is a pointer to a vector containing `nr_tspan` time steps at which to evaluate the system variables and parameter sensitivities. If `nr_tpsan` is two, the outputs are stored for each computed step.

The function `odeSD` returns the number of output values stored or any value `< 0` on error. A stack of any errors can be displayed using `errs_dump( FILE *out )`, defined in `errs.h`.

### S1.3 Automatic Generation of Functions

An important part of this work was the extraction of the necessary mathematical information used by the solvers from each model in SBML representation [3]. To this end, we developed a Matlab (MathWorks, Nantucket / MA) interface which, given an SBML model, fully automatically generates a series of suitable Matlab files that contain both the analytical representations of the system's derivatives $\mathbf{f}(t)$ and $\ddot{\mathbf{x}}(t)$ as well as the Jacobians $\mathbf{J}_f(t)$, $\mathbf{J}_{Jf}(t)$, $\partial f(t)/\partial \mathbf{p}$, and $\partial \ddot{\mathbf{x}}(t)/\partial \mathbf{p}$.

The entries of the derivative $\ddot{\mathbf{x}}(t)$ and of the matrix $\partial \ddot{\mathbf{x}}(t)/\partial \mathbf{p}$ are generated explicitly, *i.e.* without evaluating $\mathbf{J}_f(t)\mathbf{f}(t)$. The matrix $\mathbf{J}_{Jf}(t)$ is constructed as per (9), where the entries of $(\partial \mathbf{J}_f(t)/\partial \mathbf{x})\mathbf{f}(t)$ are computed explicitly and added to the square of the previously computed $\mathbf{J}_f(t)$.

The framework makes use of SBToolbox2 [4] for reading the initial SBML model and of Matlab's Symbolic Toolbox for performing various differentiations on the system's equations. The latter was preferred over parsing and differentiating the expressions in Matlab, as the Symbolic Toolbox provides the ability to simplify the sometimes clumsy or redundant expressions resulting from a straight-forward differentiation. The generated function files are written in both standard Matlab and c-language formats and can be used, in principle, with appropriate wrappers, in any solver. For example, given an SBML model in Matlab's current directory, we can write:

```
modelInfo = xml2odefun(modelname,{'c_files'}).
```

All the necessary function files in c-language format that describe the system will be generated in the current directory, and a structure providing useful information of the system, `modelInfo`, is returned.

Producing the system description files for high-dimensional (both in terms of state and parametric space) systems is a non-trivial task because the computational cost grows quadratically with the system dimension. In a fully interconnected system we will never be able to avoid such a computational cost. Remember, however, that biological systems are generally poorly interconnected, resulting in sparse Jacobians. Parsing the system's equations prior to differentiation allows us to pinpoint the elements of the various Jacobians that are non-zero and perform all computations only on these elements. In all the systems under study, the computational cost grows only linearly with the system size.

## S1.4 Framework Usage and Example

The following short Matlab code shows how to construct the integrator input files using our framework:

```
1  % Generate system files from MyModel.xml
2  curXMLfilename = 'Elowitz2000_6states.xml';
3  model_info = xml2odefun(curXMLfilename);
4
5   % generate function handles
6   mainfunctionName = 'Elowitz2000_6states';
7   jacfunctionName = 'jac_Elowitz2000_6states';
8   f = str2func(mainfunctionName);
9   df = str2func(jacfunctionName);
10
11  % take initial conditions and parameters
12  [x0 p0]=feval(f);
13
14  %set the time intervals
15  tspan = linspace(0,300,200)';
16
17  %create the options structure
18  opts = odeset( 'RelTol', 1.0e-6 , 'AbsTol' , eps , 'Jacobian' , df );
19
20  %run the integration
21  [T1 ,X1 ,~ ,~ ,~ , S1 ] = odeSD(f,tspan,x0,opts,p0);
22
23  % plot the states
24  figure (1); plot (T1 ,X1 , '.-');
25
26  % plot the sensitivities of all states w.r.t the 1st parameter
27  figure (2); plot (T1 , squeeze( S1 (: ,1 ,:) ) , '.-' );
```

We first pass the SBML model through the converter function `xml2odefun` (line 3) such that the necessary Matlab files, named `MyModel.m` and `jac_MyModel.m`, analogously to the name of the SBML file, are generated. In line 12, we extract the initial conditions for both the variables `x0` and the parameters `p0` by calling the right hand side without any arguments. The Jacobian generated by the converter is then added to an options structure (line 18) so that it can be passed to the integrator (line 21) `odeSD`, which then computes the integration and sensitivity analysis. In lines 24 and 27 we plot the system variables and the parameter sensitivities of the third variable respectively.

Analogously, the c-language files generated by the converter when called with the `'c_files'` option, can be passed to `odeSD`$_\text{c}$ as follows

```
/* Standard headers. */
#include <stdlib.h>
```

```
#include <stdio.h>
#include <string.h>
#include <math.h>
#include <float.h>

/* Local headers. */
#include "errs.h"
#include "odeSD.h"

/* Model file headers, generated by xml2odefun. */
#include "Elowitz2000_6states.h"

/* Main routine. */
int main ( int argc , char *argv[] ) {

    double *T, *X, *S, *x0;
    struct odeSD_opts opts = odeSD_opts_default;
    int i, k, nr_steps;
    double tspan[2] = { 0.0 , 300.0 };
    int nneg[ Elowitz2000_6states_Nstates ];

    /* Init x0. */
    x0 = (double *)alloca( sizeof(double) * Elowitz2000_6states_Nstates );
    memcpy( x0 , Elowitz2000_6states_x0 , sizeof(double) * Elowitz2000_6states_Nstates );
    for ( k = 0 ; k < Elowitz2000_6states_Nstates ; k++ )
        if ( x0[k] == 0.0 )
            x0[k] = DBL_EPSILON;

    /* Set some options. */
    opts.RelTol = 1.0e-6;
    opts.AbsTol = DBL_EPSILON;
    for ( k = 0 ; k < Elowitz2000_6states_Nstates ; k++ )
        nneg[k] = k;
    opts.NonNegative = nneg;
    opts.nr_NonNegative = Elowitz2000_6states_Nstates;

    /* Call the integrator on the Elowitz2000_6states problem. */
    if ( ( nr_steps = odeSD( &Elowitz2000_6states_f , &Elowitz2000_6states_jac ,
        2 , tspan , Elowitz2000_6states_Nstates , x0 , &opts ,
        Elowitz2000_6states_Nparams , Elowitz2000_6states_p , NULL ,
        &T , &X , &S ) ) < 0 ) {
        errs_dump(stderr);
        abort();
        }

    /* Print the data in gnuplot-readable format. */
    printf( "#␣t" );
    for ( k = 0 ; k < Elowitz2000_6states_Nstates ; k++ )
        printf( "␣x%02i" , k );
    printf( "\n" );
    for ( i = 0 ; i < nr_steps ; i++ ) {
        printf( "%e" , T[i] );
        for ( k = 0 ; k < Elowitz2000_6states_Nstates ; k++ )
            printf( "␣%e" , X[ i*Elowitz2000_6states_Nstates + k ] );
        printf( "\n" );
        }

    /* Clean up after odeSD. */
    free( T ); free( X ); free( S );

    /* Exit cleanly. */
    return 0;

    }
```

## S2 Accuracy of the second derivative rule and of the error estimate

To illustrate the effect of the interval width, we determined the truncation error in each step for our second-derivative rule as well as a BDF and Adams-Bashforth rule of the same degree when integrating the simple oscillator

$$\mathbf{f}(t, \mathbf{x}) = (x_2, -x_1)^{\mathsf{T}}, \quad \mathbf{x}(t_0) = (1, 0)^{\mathsf{T}}. \tag{1}$$

We employed a fixed step size of $h = 0.1$ and used the exact values of $\mathbf{x}(t) = (\sin t, \cos t)^{\mathsf{T}}$ for the previous steps in each case. As predicted, the resulting error of the second-derivative method for this test case is at least one order of magnitude lower compared to the other methods (Supporting Fig. S1A). Hence, we expect a substantial improvement of accuracy or efficiency compared to standard first-derivative methods.

Using the same test function (1) as before, we compared our new error estimate with the—computationally more efficient ($\mathcal{O}(n)$ vs. $\mathcal{O}(n^2)$)—estimate based on the difference of $\mathbf{g}_4(t + h)$ and $\mathbf{g}_5(t + h)$ over the converged solution $\mathbf{x}_1(t + h)$. The ratio of predicted to actual errors shown in Supporting Fig. S1B indicates a superior accuracy of our estimate. Furthermore, the almost uniform spacing of the errors along the $x$-axis in Figure S4 is a good indication of the robustness of the error estimate.

## S3 Results without parameter sensitivities

The results show that our integrator requires less steps than the first-derivative methods. The smaller number of steps, however, does not always translate into a speed advantage, especially for the larger models with steady-state behavior (see Fig. 1 and Supporting Fig. S2 for details). On average, the second-derivative integrator requires an approximately equal number of function evaluations (Fig. 1C), but a much larger number of evaluations of the Jacobians (Fig. 1D). This effect is a result of the instability of the second-degree rules at infinity (see Section "Methods: A second-derivative integrator") and was already observed in [5] and in [6] when studying the "ROBER" model therein. It can be understood by considering the explicit formulation of the Newton iteration matrix $\mathbf{M}(t+h)$ (8) in terms of the Jacobian $\mathbf{J}_{Jf}$ (9). The matrix $\tilde{\mathbf{J}}_f(t + h)$ is not the exact Jacobian and whatever perturbation it contains will be amplified in $(\tilde{\mathbf{J}}_f(t + h))^2$, causing the Newton iteration to converge less often than in the first-derivative rule, *i.e.* the step size is limited by variation in the second-derivative Jacobian $\mathbf{J}_{Jf}$.

This, however, is only a serious disadvantage for the models with a large number of variables and it is no disadvantage at all if the Jacobians need to be evaluated at every step, *e.g.* for very stiff systems or when computing parameter sensitivities. Integration of such models is not *infeasible*, but *inefficient* when approaching the steady state.

## References


1. Engstler C: **Matlab implementation of the Radau IIA method of order5 by Ch. Engstler after the Fortran Code RADAU5 of Hairer/Wanner** 1999. [http://na.uni-tuebingen.de/projects.shtml].

2. Shampine LF: **Implementation of implicit formulas for the solution of ODEs**. *SIAM J. Sci. Statist. Comput.* 1980, **1**:103–118.

3. Hucka M, Finney A, Sauro H, Bolouri H, Doyle J, Kitano H, Arkin A, Bornstein B, Bray D, Cornish-Bowden A, Cuellar A, Dronov S, Gilles E, Ginkel M, Gor V, Goryanin I, Hedley W, Hodgman T, Hofmeyr J, Hunter P, Juty N, Kasberger J, Kremling A, Kummer U, Novere NL, Loew L, Lucio D, Mendes P, Minch E, Mjolsness E, Nakayama Y, Nelson M, Nielsen P, Sakurada T, Schaff J, Shapiro B, shimizu T, Spence H, Stelling J, Takahashi K, Tomita M, Wagner J, Wang J: **The Systems Biology Markup Language (SBML): a medium for representation and exchange of biochemical network models**. *Bioinformatics* 2003, **19**(4):524–31.

4. Schmidt H, Jirstrand M: **Systems Biology Toolbox for MATLAB: a computational platform for research in systems biology**. *Bioinformatics* 2006, **22**(4):514–5. [Schmidt, Henning Jirstrand,

Mats Research Support, Non-U.S. Gov't England Bioinformatics (Oxford, England) Bioinformatics. 2006 Feb 15;22(4):514-5. Epub 2005 Nov 29.].

5. Enright WH: **Second Derivative Multistep Methods for Stiff Ordinary Differential Equations**. *SIAM J. Numer. Anal.* 1974, **11**(2):321–331.

6. Hairer E, Norsett S, Wanner G: *Solving Ordinary Differential Equations I.* Berlin: Springer Verlag 1987.

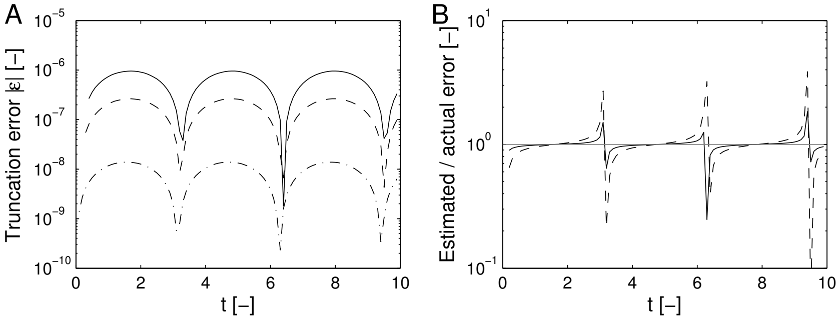


Supporting Figure S1: Numerical errors and error estimation for the simple oscillator example (1). (**A**) Truncation errors of the second-derivative formula in (7) (dash-dotted line), of a BDF (solid line), and of an Adams-Moulton (dashed line) formula of the same degree for the values of $x_1$ with a constant step size $h = 0.1$. (**B**) Ratio of the predicted vs. the exact error of $x_1$ with $h = 0.2$ for the 'traditional' error estimate using the difference between two approximations of different degrees yet computed over the same nodes, $g_4(t + h) - g_5(t + h)$, (dashed line) and for the new error estimate in (11) (solid line).

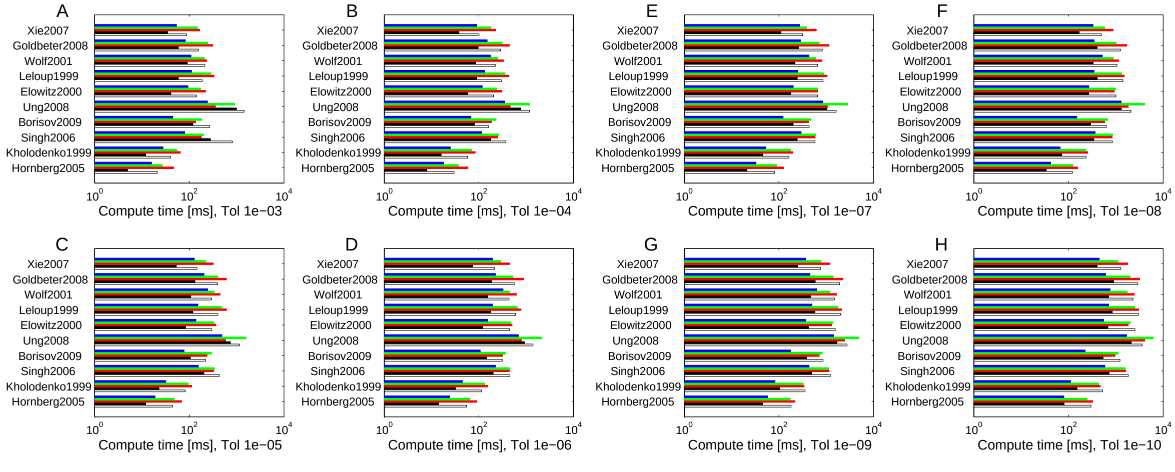


Supporting Figure S2: Detailed performance comparison without parameter sensitivities. All models were integrated for the time spans shown in Table 2. (**A-H**) Computation times for the individual models listed in Table 2 with varying relative tolerances (see X-axis labels) using `odeSD` (white bars), $\texttt{odeSD}_{\texttt{mex}}$ (black), `ode15s` (red), `radau5` (green), and `cvodes` (blue).

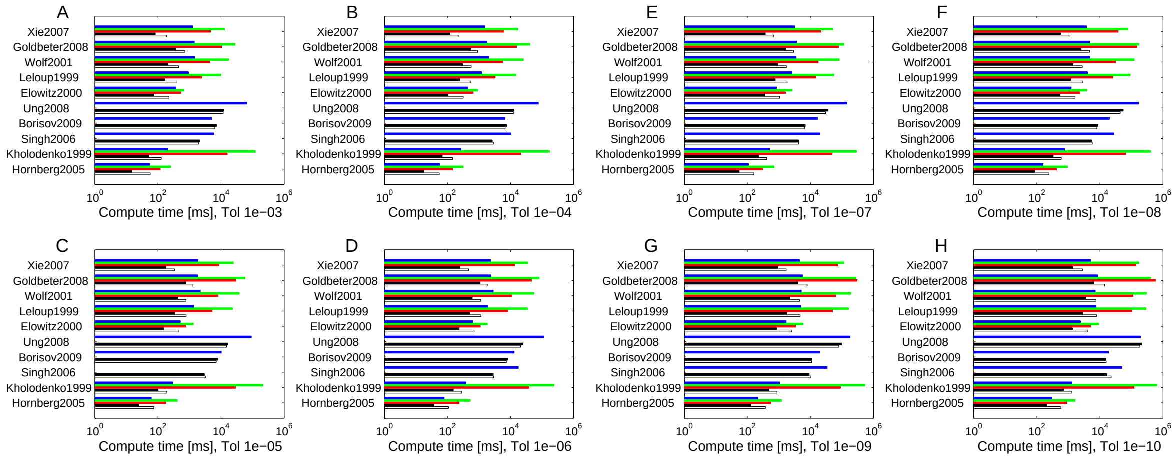


Supporting Figure S3: Detailed performance comparison with parameter sensitivities. All models were integrated for the time spans shown in Table 2. (**A-H**) Computation times for the individual models listed in Table 2 with varying relative tolerances (see X-axis labels) using `odeSD` (white bars), $\texttt{odeSD}_{\texttt{mex}}$ (black), `ode15s` (red), `radau5` (green), and `cvodes` (blue).

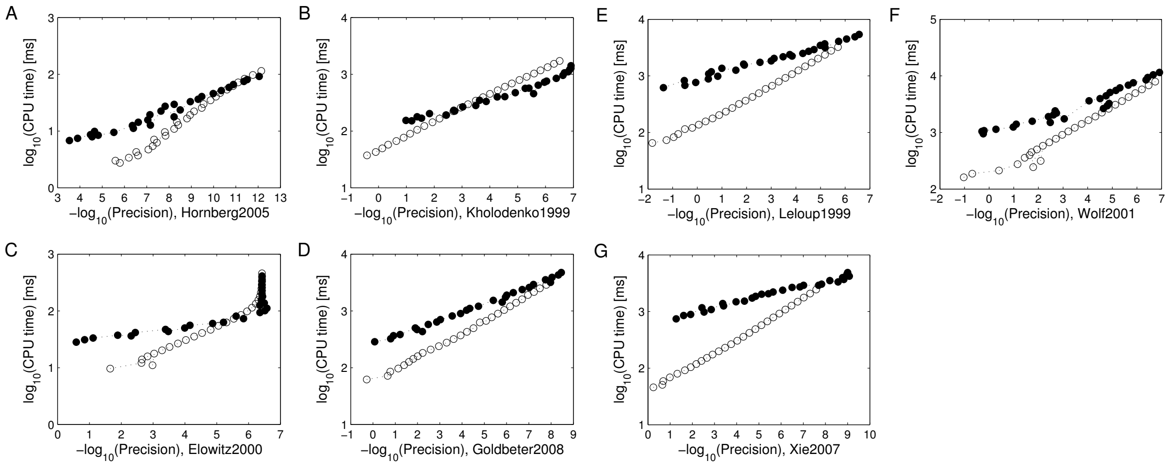


Supporting Figure S4: Precision-work diagrams for the c-language version $\texttt{odeSD}_{\texttt{c}}$ (open black circles) and `cvodes` (filled black circles) with parameter sensitivities. (**A-G**) Computation times for all models for which the systems dynamics were solved with all ODE integrators (see X-axis for model specifications) as a function of precision. The models were integrated for the time spans shown in Table 2.
